# Proteomic Analyses of Clots Identify Stroke Etiologies in Patients Undergoing Endovascular Therapy

**DOI:** 10.1111/cns.70340

**Published:** 2025-03-13

**Authors:** Tae Jung Kim, Jin Woo Jung, Young‐Ju Kim, Byung‐Woo Yoon, Dohyun Han, Sang‐Bae Ko

**Affiliations:** ^1^ Department of Neurology Seoul National University College of Medicine Seoul Korea; ^2^ Department of Critical Care Medicine Seoul National University Hospital Seoul Korea; ^3^ Transdisplinary Department of Medicine & Advanced Technology Seoul National University Hospital Seoul Korea; ^4^ Proteomics Core Facility Biomedical Research Institute, Seoul National University Hospital Seoul Korea; ^5^ Department of Neurology Uijeongbu Eulji Medical Center Uijeongbu Korea

**Keywords:** biomarker, clot, proteomic analysis, stroke mechanisms

## Abstract

**Aims:**

This study aimed to investigate the correlation between clot composition and stroke mechanisms in patients undergoing endovascular therapy (EVT), using proteomic analysis.

**Methods:**

This study included 35 patients with ischemic stroke (cardioembolism [CE], *n* = 17; large artery atherosclerosis [LAA], *n* = 6; cancer‐related [CR], *n* = 4; and undetermined (UD) cause, *n* = 8) who underwent EVT. Retrieved clots were proteomically analyzed to identify differentially expressed proteins associated with the three stroke mechanisms and to develop the machine learning model.

**Results:**

In the discover stage, 3838 proteins were identified using clot samples from 27 patients with CE, LAA, and CR mechanisms. Through functional enrichment and network analysis, 149 proteins were identified as potential candidates for verification studies. After verification experiments, 34 proteins were selected as the final candidates to predict stroke mechanisms. Furthermore, the machine learning‐based model identified three proteins associated with each mechanism (Pleckstrin in CE; CD59 glycoprotein in LAA; and Immunoglobulin Heavy Constant Gamma 1 in CR) in the UD group.

**Conclusions:**

This study identified specific protein markers of clots that could differentiate stroke mechanisms in patients undergoing EVT. Therefore, our results could offer valuable insights into elucidating the mechanisms of ischemic stroke, which could provide information on more effective secondary prevention strategies.

## Graphical abstract

We successfully identified specific biomarkers associated with stroke mechanisms. We developed a methodology that enabled the identification of biomarkers for different stroke subtypes, such as cardioembolism, large artery atherosclerosis, and cancer‐related, through proteomic analysis.

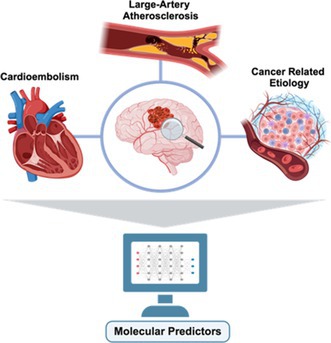

## 1 Introduction

Approximately 25% of patients experiencing ischemic stroke face the grim prospect of recurrence. Therefore, a strategy for secondary prevention is to define the underlying cause and mechanism of ischemic stroke, enabling the identification of targets to mitigate the risk of recurrence [1, 2]. Endovascular therapy (EVT) has been the standard of care in patients with ischemic stroke and large vessel occlusion (LVO) [3]. Thus, meticulous analysis of clot characteristics and composition could be useful for identifying stroke mechanisms and etiologies. Assessing the composition of retrieved clots would help differentiate stroke etiology and better inform secondary prevention strategies after ischemic stroke. Previous studies employing histological analysis have demonstrated that the composition of red blood cells (RBCs), white blood cells (WBCs), and fibrin/platelets correlates with resistance to intravenous thrombolysis (IVT) or EVT in patients with LVO [3, 4, 5, 6]. Furthermore, RBC‐rich red thrombi and fibrin‐rich white thrombi have been associated with cardioembolism (CE) and large artery atherosclerosis (LAA), respectively [5]. Other studies have reported contrasting results or no differences concerning stroke etiologies [3, 4, 6]. While some studies have demonstrated an association between clot composition and stroke etiologies, there remains insufficient evidence of the correlation between the proteins or biomarkers constituting a clot and the etiologies of ischemic stroke [4, 5, 6]. Analyzing the compositions of retrieved clots from occlusive cerebral arteries may provide valuable insights for differentiating the etiologies of ischemic stroke. Therefore, this study aimed to characterize protein expression patterns through proteomic analyses of retrieved clots and identify key protein biomarkers based on distinct stroke etiologies.

## 2 Methods

### 2.1 Study Populations and Clinical Information

We prospectively included patients with ischemic stroke due to LVO in the intracranial internal carotid artery (ICA), middle cerebral artery (MCA), and basilar artery (BA) who underwent EVT between July 2017 and September 2019. Inclusion criteria are as follows: (1) age > 18 years, (2) confirmed LVO in the arteries mentioned above using brain CT angiography or brain MR angiography prior to EVT, and (3) an adequate amount of retrieved clots for proteomic analysis. Clot specimens obtained during EVT procedures were stored at an appropriate temperature of −80°C for analysis. During the study period, clot samples were collected from a total of 44 patients. However, nine samples were excluded from the proteomics analysis due to storage issues (i.e., not being immediately stored in a liquid nitrogen tank after clot retrieval). Successful angiographic recanalization was defined by a Thrombolysis in Cerebral Infarction (TICI) score of 2b or 3 at the end of EVT [7]. We collected the following clinical data from the patients included in this study: demographic information, body weight (Kg), body mass index (Kg/m^2^), laboratory data related to thrombosis such as platelet count, coagulation panel, liver function, and renal function, and vascular risk factors; stroke severity according to National Institute of Health Stroke Scores (NIHSS) on admission and at discharge; clinical information related to reperfusion therapy such as IVT and EVT; prior use of antiplatelet or anticoagulant medications; and presence of active cancer. Furthermore, any medications taken prior to the index stroke that might affect thrombosis were analyzed, including nonsteroidal anti‐inflammatory drugs (NSAIDs), antidepressants, steroid, and hormone therapy. The mechanisms of ischemic stroke were determined at the time of discharge as LAA, CE, cancer‐related (CR), and undetermined (UD) with negative cause according to the classification of the Trial of Org 10,172 in acute stroke treatment [8]. Finally, a total of 35 patients who had successful recanalization after EVT were included as follows for this study: 6 with LAA, 17 with CE, 4 with CR, and 8 with UD causes. Among them, 27 patients with LAA, CE, and CR mechanisms were selected for the analysis. A validation study of the model developed for identifying stroke mechanisms was conducted using clots from the eight patients with an UD cause. This study was reviewed and approved by the Institutional Review Board of our institution (No. H‐1805‐050‐944 and H‐1803‐078‐931), and the participants or family provided written informed consent. Additionally, all methods of this study were conducted according to the regulation of the Declaration of Helsinki and guidelines.

### 2.2 Clots Collection

EVT was performed according to the standard protocols and procedures of our institution. After EVT, clots were carefully detached from the retriever devices and preserved at −196°C in liquid nitrogen for 30 min until further analysis.

### 2.3 Experimental Design and Statistical Rationale

Discovery study included 35 clot samples (*n* = 17 in CE, *n* = 6 in LAA, *n* = 4 in CR, and *n* = 8 in UD) for identification of specific proteins according to stroke mechanisms. A total of 35 biological replicates and one technical replicate were analyzed in the discovery stage. After preprocessing for accurate relative quantification, multiple group comparison analysis was performed to identify the group‐specific expressed proteins using ANOVA with a normal *p*‐value < 0.05. To assess the consistency of group‐specific expressed proteins, 27 clot samples consisting of CE (*n* = 17), LAA (*n* = 4), and CR (*n* = 8) were analyzed with one technical replicate using DIA methods as a verification phase. Additionally, verified group‐specific proteins were evaluated by multiple testing correction of the discovery data at the 15% FDR level. Furthermore, intra‐arterial blood samples were drawn before and after EVT at the proximal site of the occluded vessel, and 10 pre‐EVT serums and 10 post‐EVT serums from 10 patients were analyzed. A flowchart for data processing, including statistical analysis, was described in Figure S1. Detailed methods for histologic and proteomic analysis of clots were discussed in Data S1. Moreover, detailed statistical analysis and bioinformatics analysis were presented in Data S1.

### 2.4 Developing, Training, and Testing Models for the Selection of Stroke Mechanisms

We assessed the discriminatory power of the validated differentially expressed proteins (DEPs) using data‐dependent acquisition (DDA) and data‐independent acquisition (DIA) analyses. Among DEPs, we identified proteins that exhibited equivalent expression patterns in both analyses. The selected proteins were used to construct a model to predict stroke mechanisms in the UD group. The DIA data set was divided into a training set, comprising 70% of the data, for constructing the prediction model. The remaining data were classified as the test set and used to validate the model. To ensure accurate selection and avoid overfitting during model development, the entire dataset was tuned using 10‐fold cross‐validation within the training set. Random forest (RF) feature selection was employed to determine the variable importance among the selected DEPs from the training set. Subsequently, a support vector machine (SVM) model was used to predict the conditions of the testing set, and the results were applied to the UD group data set. Before feature selection, automated machine‐learning (AutoML) hyperparameter tuning was applied to enhance the performance of the model [9]. The protein expression of the final selected feature candidates was transformed using the log2 function to develop the model. An H_2_O machine learning platform was used to facilitate hyperparameter tuning. This platform provides parallelized implementations of several supervised machine learning algorithms, including RF and generalized linear models. The selected features were filtered at the 90th percentile threshold to select the top 10% of features that would have a significant impact on the accuracy of the developed model.

## 3 Results

### 3.1 Baseline Characteristics and Clinical Information

Out of 35 patients, 27 (mean age, 73.3 years; male, 37.0%) were selected for analysis of clots based on stroke mechanisms. The median NIHSS score upon admission was 19 (IQR, 11–20). Prior to EVT, seven patients (24.9%) received IVT (Table 1). Regarding the locations of the occluded vessels and the occlusive sites were distributed as follows: ICA (33.3%), MCA (59.3%), and BA (7.4%). Initial NIHSS was significantly higher in cancer‐related group. Additionally, Prothrombin Time and International Normalized Ratio, and d‐dimer levels were significantly elevated in the CR group. Regarding previous medications, none of the patients had received steroids or hormone therapy. Additionally, no statistically significant differences were observed in the use of NSAIDs, antidepressants, antiplatelet agents, and anticoagulants across the three groups (Table 1). The laboratory findings indicate that patients with CR stroke exhibited elevated levels of D‐dimer, as well as prolonged Prothrombin Time and International Normalized Ratio values, in comparison to other stroke mechanism groups (Table 1). In the analysis of clots using H&E staining, there were no differences in the relative proportions of RBCs, fibrin, and leukocytes among the three groups (Table 1 and Figure S2). When comparing the clinical information between the initially included patients and those with UD causes (*n* = 8), the UD cause group showed significantly lower proportions of atrial fibrillation (AF) and patients with functional independence before their stroke (Table S1).

**TABLE 1:** Baseline characteristics of the study population.

	Total (*n* = 27)	CE (*n* = 17, 63.05)	LAA (*n* = 6, 22.2%)	CR (*n* = 4, 14.8%)	*p*
Age, mean (SD), y	73.3 (12.9)	77.7 (8.1)	68.7 (14.6)	61.3 (19.8)	0.037
Sex (Male), *n* (%)	10 (37.0)	5 (29.4)	3 (50.0)	2 (50.0)	0.612
Body weight, mean (SD), Kg	56.9 (11.8)	56.2 (12.8)	56.0 (5.1)	61.3 (16.3)	0.737
BMI, mean (SD), Kg/m^2^	22.1 (3.5)	22.1 (3.9)	22.2 (2.4)	22.1 (4.1)	0.998
HT, *n* (%)	17 (63.0)	11 (64.7)	4 (66.7)	2 (50.0)	0.862
DM, *n* (%)	10 (37.0)	4 (23.5)	5 (83.3)	1 (25.0)	0.034
DL, *n* (%)	6 (22.2)	2 (11.8)	3 (50.0)	1 (25.0)	0.080
Previous Stroke Hx. *n* (%)	5 (18.5)	4 (23.5)	1 (20.0)	0 (0.0)	0.798
Coronary artery disease, *n* (%)	3 (11.1)	2 (11.8)	1 (16.7)	0 (0.0)	1.000
A.fib, *n* (%)	17 (63.0)	17 (100.0)	0 (0.0)	0 (0.0)	< 0.001
Smoking, *n* (%)	5 (18.5)	3 (17.6)	2 (33.3)	0 (0.0)	0.621
Active cancer, *n* (%)	4 (14.8)	0 (0.0)	0 (0.0)	4 (100.0)	< 0.001
Initial NIHSS, median (IQR)	19 (16–23)	18.0 (14.5–21.0)	19.5 (15.0–23.0)	24.0 (22.3–25.0)	0.036
Discharge NIHSS, median (IQR)	8 (3–15)	6.0 (4.0–16.0)	5.0 (1.8–13.5)	12.5 (10.5–17.5)	0.076
Prestroke mRS = 0, *n* (%)	23 (85.2)	14 (82.4)	6 (100.0)	3 (75.0)	0.582
Lesion, *n* (%)					
MCA	16 (59.3)	11 (64.7)	2 (33.3)	3 (75.0)	0.162
ICA	9 (33.3)	6 (35.3)	2 (33.3)	1 (25.0)	
BA	2 (7.4)	0 (0.0)	2 (33.3)	0 (0.0)	
IV thrombolysis, *n* (%)	7 (25.9)	5 (29.4)	0 (0.0)	2 (50.0)	0.148
Prior antiplatelet agents, *n* (%)	7 (25.9)	4 (23.5)	3 (50.0)	0 (0.0)	0.201
Prior anticoagulants, *n* (%)	4 (14.8)	3 (17.6)	0 (0.0)	1 (25.0)	0.582
Prior NSAID, *n* (%)	1 (3.7%)	1 (5.9)	0 (0.0)	0 (0.0)	1.000
Prior antidepressants, *n* (%)	3 (11.1%)	3 (17.6)	0 (0.0)	0 (0.0)	0.721
Onset to ER visit time (min), median (IQR)	50.0 (36.0–180.0)	57.0 (37.5–117.0)	43.5 (31.0–230.5)	137.5 (25.3–258.8)	0.708
Onset to rt‐PA time (min), median (IQR)	95.0 (60.0–100.0)	95.0 (74.5–120.5)	N/A	78.5 (59.0‐ N/A)	1.000
Onset to reperfusion time (min), median (IQR)	210.0 (180.0–320.0)	201.0 (179.0–313.0)	231.0 (117.5–386.8)	261.0 (165.0–393.0)	0.560
Laboratory information, mean (SD)					
WBC (×1000)/uL	9.13 ± 4.37	8.59 ± 4.10	9.82 ± 1.00	10.4 ± 8.33	0.712
Hb, g/dL	12.6 ± 2.2	12.8 ± 1.8	13.1 ± 1.8	10.9 ± 4.0	0.280
PLT (×1000)/uL	207.9 ± 79.6	213.8 ± 73.6	217.8 ± 84.3	168.0 ± 108.3	0.570
PT‐INR	1.05 ± 0.12	1.02 ± 0.06	1.00 ± 0.05	1.23 ± 0.22	0.001
aPTT, sec	29.5 ± 3.4	29.6 ± 2.9	28.5 ± 2.2	30.6 ± 6.6	0.635
D‐dimer, ug/mL	3.37 ± 4.81	2.23 ± 2.22	1.47 ± 1.38	11.02 ± 8.84	0.001
Initial glucose, mg/dL	164.0 ± 67.4	147.8 ± 34.1	147.8 ± 55.9	179.3 ± 153.8	0.248
BUN, mg/dL	16.3 ± 6.5	15.8 ± 5.4	19.8 ± 8.7	12.8 ± 6.2	0.222
Cr, mg/dL	1.03 ± 0.60	1.08 ± 0.66	1.13 ± 0.57	0.70 ± 0.26	0.489
AST, IU/L	24.9 ± 6.4	25.7 ± 6.8	21.5 ± 4.8	26.8 ± 6.0	0.324
ALT, IU/L	19.2 ± 8.3	19.8 ± 9.0	19.8 ± 7.1	15.8 ± 7.5	0.684
Ca, mg/dL	8.8 ± 0.5	8.9 ± 0.4	8.7 ± 0.3	8.3 ± 0.6	0.066
Clot proportion in H&E stain, *n* (%)					
RBC	40.3 ± 26.1	39.4 ± 21.7	33.4 ± 12.2	49.0 ± 47.3	0.734
Fibrin	55.5 ± 25.5	56.1 ± 21.5	62.3 ± 14.0	47.9 ± 45.7	0.767
Leukocytes	4.1 ± 1.8	4.4 ± 1.7	4.7 ± 1.5	3.0 ± 2.2	0.361

Abbreviations: A.fib, atrial fibrillation; ALT, alanine aminotransferase; aPTT, Activated Partial Thromboplastin Time; AST, aspartat aminotransferase; BA, basilar artery: emergency room; BUN, blood urea nitrogen; Ca, calcium; CE, cardioembolism; CR, cancer‐related; Cr, creatinine; DL, dyslipidemia; DM, diabetes mellitus; H & E, Hematoxylin and Eosin; Hb, hemoglobin; HT, hypertension; ICA, intracranial carotid artery; IQR, interquartile range; LAA, large artery atherosclerosis; MCA, middle cerebral artery; mRS, modified Rankin Scale; NIHSS, National Institutes of Health Stroke Scale; PLT, platelet; PT‐INR, Prothrombin Time and International Normalized Ratio; RBC, red blood cell; rt‐PA, recombinant tissue plasminogen activator; SD, standard deviation; WBC, white blood cell.

### 3.2 Quantitative Proteome Analysis of Clot Samples Based on Stroke Mechanisms

**FIGURE 1:**
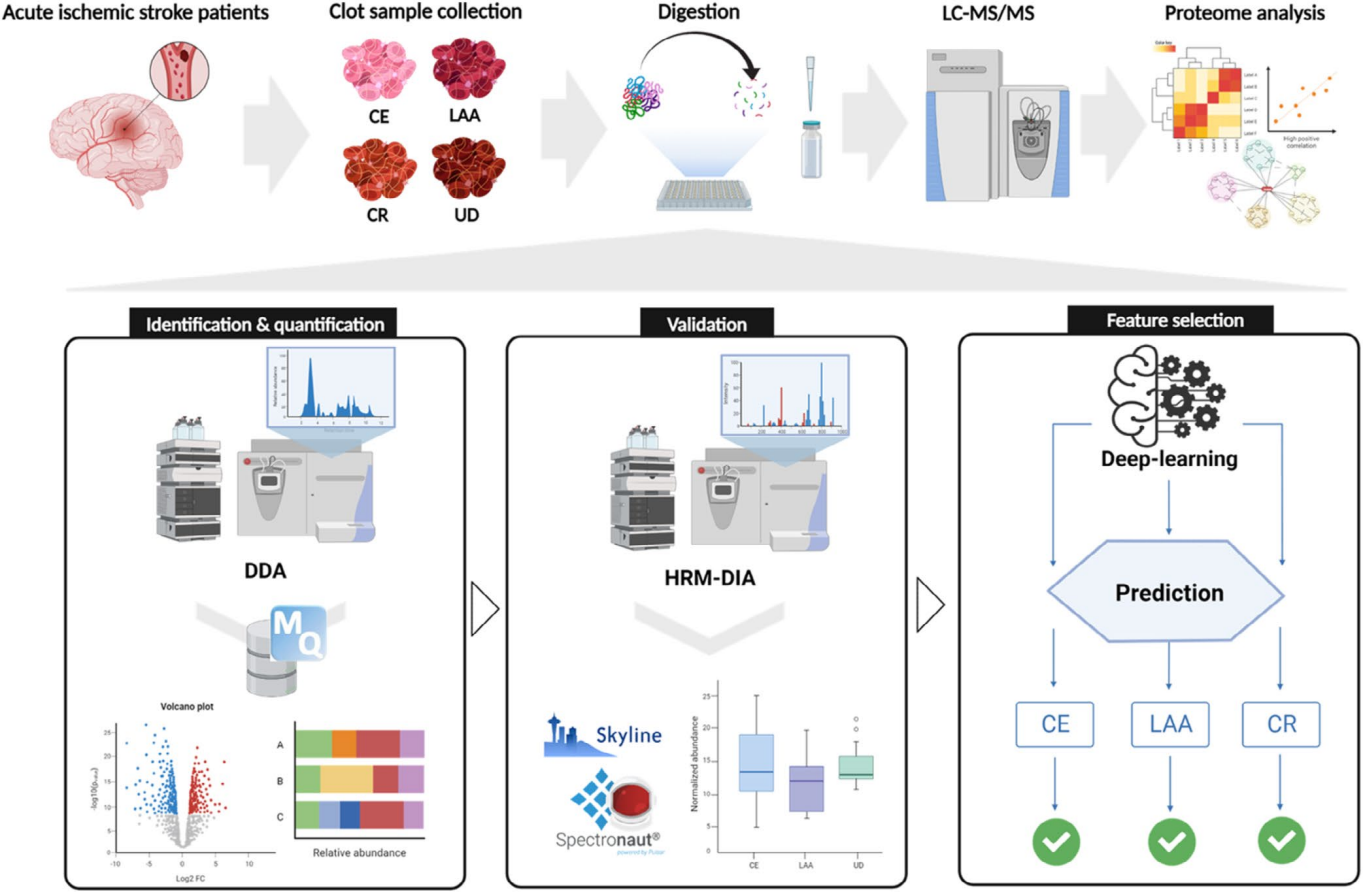
Overall scheme of analysis. This figure was created with Biorender.com and exported under a paid subscription.

For global proteome profiling, clot samples were analyzed from four groups including CE (*n* = 17), LAA (*n* = 6), CR (*n* = 4), and UD (*n* = 8). The analysis scheme is presented in Figure 1 and Figure S1. To obtain an in‐depth proteome of clot samples without depletion of high‐abundant blood proteins, we used a combination of peptide prefractionation and a matching library of pooled clot. In total, 3839 proteins were identified at FDR < 1% (Table S2). Among these, an average 1944, 1851, 2109, and 1981 proteins were quantified in the CE, LAA, CR, and UD groups, respectively (Figure S3A and Table S2). The reproducibility of global proteome profiling was evaluated by calculating the coefficient of variation (CV) in the quantification of proteins throughout all LC–MS/MS runs in each sample group, indicating that our label‐free quantification shows high reproducibility (Figure S3B). As expected, blood proteins were observed as highly abundant proteins in dynamic ranges of the clot proteome that spanned seven orders (Figure S3C–E).

To discover protein signatures among sample groups according to determined stroke mechanisms, we first performed statistical analysis using three groups, including CE, LAA, and CR. A total of 2573 proteins were identified in common, occupying 74.0% of total identified proteins in the three groups (Figure S4A). Principal component analysis (PCA) revealed a degree of separation between the LAA and CR groups, although some overlap with the CE group was observed. The CE group's distribution suggests potential heterogeneity in protein expression within this group, as it overlaps with both LAA and CR groups (Figure S4B). Using proteins with 70% of valid values (Table S3), a total 260 DEPs were identified among the three groups based on ANOVA multiple group comparison test (Table S4). Additionally, hierarchical clustering using 260 DEPs resulted in the identification of three distinct expression clusters (Cluster 1,2, and 3), showing the increased expression of proteins associated with each stroke mechanism in comparison to other stroke mechanisms (Figure S4C). Cluster 1 represents the upregulation of proteins from LAA groups among the three groups, whereas cluster 2 represents the upregulation of proteins from CE groups. Upregulation of proteins from CR groups was observed in cluster 3.

### 3.3 Functional Enrichment and Protein Interaction Analysis After Hierarchical Clustering According to Stroke Mechanisms

To investigate molecular pathophysiology according to three different stroke etiologies, gene ontology (GO) analysis was conducted on each cluster. Although several biological processes including protein metabolic processes, immune response, and actin filament‐related processes underlie in more than one stroke etiology due to the complexity of the cerebrovascular disease, many biological processes are supposed to play a role in the pathophysiology of each stroke subtype. For example, in cluster 1 (LAA specific), 79 proteins that are known to be associated with processes such as ubiquitination, the ubiquitin‐proteasome system, endothelial migration, and atheroma formation were significantly upregulated (Figure S5A and Table S5). In cluster 2 (CE specific), many proteins were significantly enriched to several terms related to fibrillation, such as actin cytoskeleton organization, supramolecular fiber organization, and platelet aggregation (Figure S5C and Table S6). Hemostasis, coagulation, apoptotic processes, and tricarboxylic acid cycle were also enriched, which are associated with major cardiovascular events including AF, thrombosis, cardiomyopathy, and myocardial infarction [10, 11, 12, 13]. In the CR specific group (cluster 3), 37 proteins that are associated with cancer markers, tumorigenesis, and immune systems, including the complement system, were significantly upregulated (Figure S5E and Table S7).

By integrating these GO terms, 11 modules were obtained in cluster 1, which are mostly associated with protein metabolic process regulation (module 1), actin depolymerization complex (module 2), and response to metal homeostasis (module 3) (Figure S6A). In cluster 2, 13 modules were enriched into biological processes mainly related to cellular macromolecule protein (module 1), ameboidal cell adhesion (module 2), and hydrolase involvement in apoptosis (module 3) (Figure S6B). Only three modules were obtained in cluster 3, consisting of immune response activation (module 1), protein activity regulation (module 2), and complement activation alternative (module 3).

Next, only GO terms that can be supported by the experimental evidence or literature were manually picked to narrow down the list of protein candidates. In each cluster, the proteins included in the selected GO terms were mapped to a list of proteins, showing that 62, 57, and 30 proteins were identified as potential candidates for predictive features and validation studies in the LAA, CE, and CR groups, respectively (Table S8). To assess our selection of protein candidates, we constructed a protein–protein interaction (PPI) network in each cluster. The candidate proteins in LAA‐specific group were mainly related to the regulation of protein metabolic processes, the regulation of immune system processes, and protein ubiquitination (Figure S5B). Interestingly, main hub proteins (SNCA, SLC4A1, and EPB42) were mainly related to lipid pathways and inflammation that play an emerging role in LAA [14]. In the case of the CE group, 57 proteins were mainly involved in the regulation of hemostasis, vesicle‐mediated transport, apoptotic signaling processes, and small GTPase‐mediated signal transduction (Figure S5D). ACTB, TLN1, PLEK, ROCK2, SRC, and CSK were observed as main hub proteins, which are closely associated with coagulation, hemostasis, and thrombosis [15]. Remarkably, main hub proteins in the CR group were VTN, C3, C4B, C9, and CFH that play a major role in the complementary pathway (Figure S5F), which has gained increased attention as a major contributor to cancer‐related coagulopathy [16].

### 3.4 HRM‐DIA Verification Phase

Potential candidates selected from functional enrichment analysis were verified using orthogonal methods. Using spiked‐in 11 iRT peptides, the DIA acquisition and targeted data analysis were performed with retention‐time‐normalized spectral libraries [17]. The HRM‐DIA experiments resulted in the quantification of 1043 proteins (Table S9) in a total 27 clot samples. We performed ANOVA pattern clustering analysis of differentially expressed proteins related to three stroke mechanisms with ANOVA *p*‐value < 0.05. The hierarchical cluster revealed that three clusters are observed with a pattern of upregulated proteins, with 71, 357, and 6 proteins in cluster 1 (LAA specific), cluster 2 (CE specific), and cluster 3 (CR specific), respectively (Figure S7 and Table S10).

**FIGURE 2:**
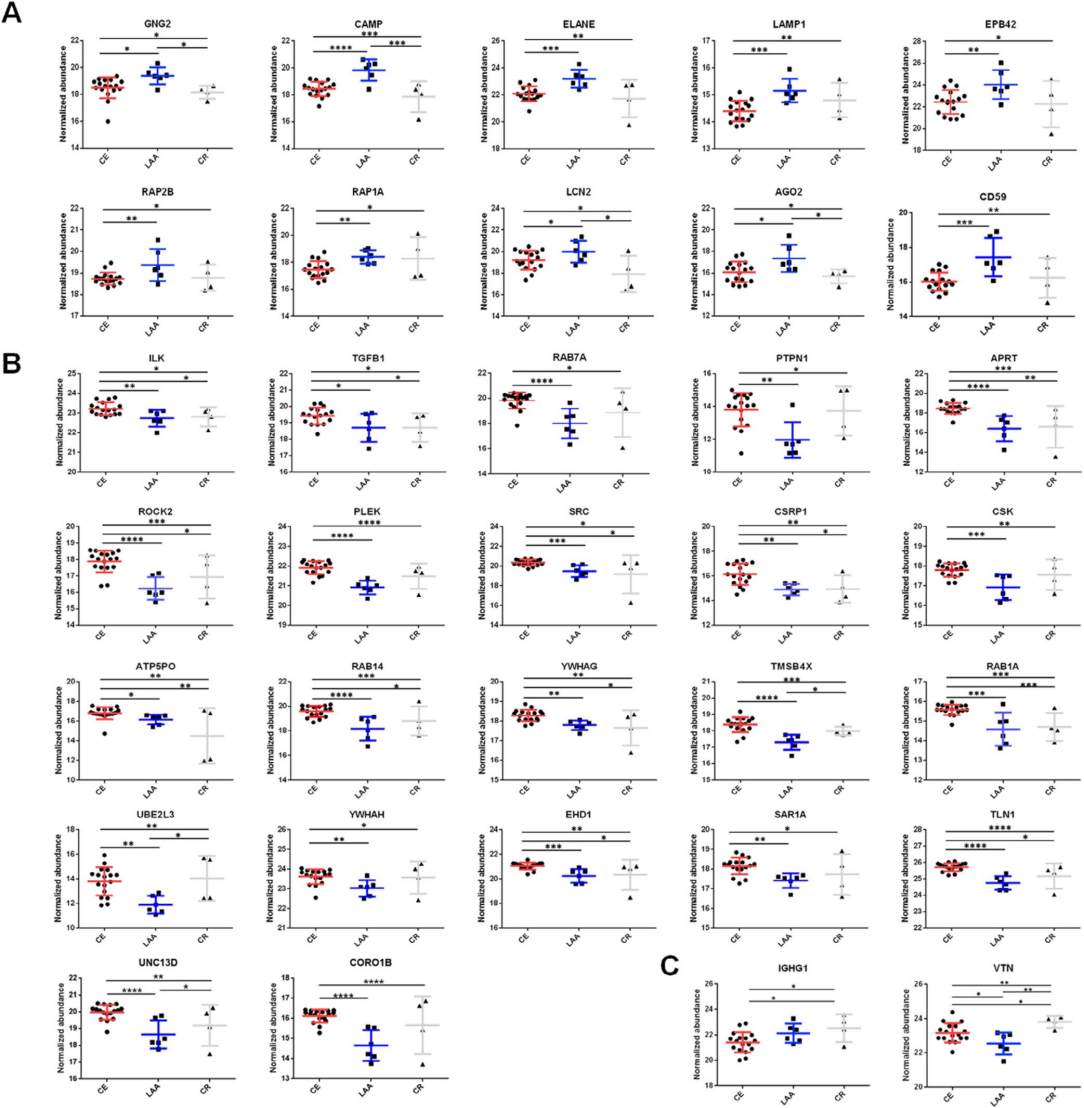
Expression patterns of final candidates for feature selection. Dot plot showing expression level of final candidate proteins among three stroke mechanism groups. The mean with standard deviations is marked as line and distinguish it by different colors. Final candidates consisted of 10, 22, and 2 proteins, in the LAA (A), CE (B), and CR (C) group, respectively. The statistical significance from student *t*‐test are also marked with asterisks (*p < 0.05, **p < 0.01, ***p < 0.001, and ****p < 0.0001).

In comparison with discovery data, 10, 23, and 2 proteins were commonly identified in cluster 1, 2, and 3, respectively (Figure S7 and Table S10). Additional pair‐wise analyses were performed to compare significant individual expression changes (Table 2). Finally, 34 proteins were selected as the final candidates for feature selection, except for the PRDX6 protein, which showed no significant *p*‐value in pair‐wise comparisons. Expression patterns for 10, 22, and 2 proteins in LAA, CE, and CR specific groups were shown as Figure 2. These proteins were subsequently used for deep learning feature selection. Additionally, results of blood samples in CE were shown in the Data S2. Especially, we found that RAB1A, TLN1, UBE2L3, and YWHAH were included in the final blood protein candidates related to the CE mechanism. Identified proteins from arterial blood were found to have increased expression in the clots of the CE mechanism group (Table S13).

**TABLE 2:** The statistical significance and expression difference from ANOVA and *t* test between each group of final prediction feature candidates.

	ANOVA		T‐test								
Cluster	Gene name	*p*	Log2FC (CE vs. LAA)	*p*	Sig	Log2FC [CE vs. CR]	*p*	Sig	Log2FC (LAA vs. CR)	*p*	Sig
CE specific	ILK	0.0156	0.4915	0.0071	**	0.4098	0.0481	*	0.5897	0.7863	—
	TGFB1	0.0344	0.7267	0.0205	*	0.7146	0.0391	*	0.0121	0.9833	—
	RAB7A	0.0029	1.8130	0.0001	****	0.9600	0.0901	—	0.8527	0.4039	—
	PTPN1	0.0059	1.8510	0.0012	**	0.0882	0.8879	—	−1.7630	0.0622	—
	APRT	0.0003	2.0540	0.0001	****	1.8790	0.0027	**	−0.1750	0.8733	—
	ROCK2	0.0005	1.6390	0.0001	****	0.9382	0.0489	*	−0.7005	0.2987	—
	PLEK	0.0001	1.0020	0.0001	****	0.4321	0.0658	—	−0.5696	0.1033	—
	SRC	0.0137	0.8579	0.0002	***	1.1660	0.0196	*	0.3082	0.7192	—
	CSRP1	0.0045	1.2470	0.0033	**	1.2100	0.0272	*	0.0369	0.9420	—
	CSK	0.0039	0.8740	0.0004	***	0.2447	0.3276	—	−0.6293	0.2007	—
	ATP5PO	0.0048	0.6468	0.0302	*	2.3060	0.0037	**	1.6600	0.1850	—
	RAB14	0.0010	1.4030	0.0001	****	0.7642	0.0390	*	−0.6390	0.3698	—
	YWHAG	0.0090	0.4911	0.0014	**	0.6322	0.0189	*	0.1411	0.7149	—
	TMSB4X	0.0001	1.0700	0.0001	****	0.3829	0.1203	—	−0.6866	0.0292	*
	RAB1A	0.0004	0.9920	0.0002	***	0.8751	0.0005	***	0.1169	0.8263	—
	UBE2L3	0.0065	1.9010	0.0012	**	−0.2373	0.7443	—	−2.1390	0.0304	*
	YWHAH	0.0411	0.5888	0.0045	**	0.0401	0.8812	—	−0.5487	0.1940	—
	EHD1	0.0072	0.7949	0.0001	***	0.7175	0.0301	*	−0.0770	0.8934	—
	SAR1A	0.0208	0.7361	0.0012	**	0.4255	0.1933	—	−0.3106	0.5095	—
	TLN1	0.0001	0.9632	0.0001	****	0.5511	0.0164	*	−0.4121	0.2963	—
	CORO1B	0.0048	1.4632	0.0001	****	0.4557	0.2183	—	−1.0082	0.1823	—
	UNC13D	0.0011	1.3212	0.0001	****	0.0370	0.7853	*	−0.5350	0.4303	—
LAA specific	GNG2	0.0040	−0.8930	0.0215	*	−0.3392	0.4259	—	1.2320	0.0116	*
	CAMP	0.0023	−1.3970	0.0001	****	−0.5710	0.1459	—	1.9680	0.0125	*
	ELANE	0.0058	−1.1200	0.0006	***	−0.3437	0.4219	—	1.4640	0.0536	—
	LAMP1	0.0035	−0.7563	0.0005	***	0.4041	0.1059	—	0.3522	0.3283	—
	EPB42	0.0415	−1.6070	0.0082	**	−0.1873	0.8004	—	1.7940	0.1365	—
	RAP2B	0.0260	−0.6388	0.0061	**	0.0464	0.8183	—	0.5924	0.2236	—
	RAP1A	0.0296	−0.9426	0.0032	**	0.8241	0.0971	—	0.1185	0.8638	—
	LCN2	0.0191	−0.7973	0.0831	—	−1.2820	0.0424	*	2.0790	0.0405	*
	AGO2	0.0205	−1.2720	0.0167	*	−0.4061	0.4303	—	1.6780	0.0423	*
	CD59	0.0033	−1.414	0.0004	***	−0.2206	0.5603	—	1.1940	0.1397	*
CR specific	IGHG1	0.0362	−0.7187	0.0682	—	−1.1210	0.0284	*	−0.4026	0.5013	—
	VTN	0.0064	0.6200	0.0348	*	−0.6336	0.0446	*	−1.2540	0.0075	**

*Note:* Sig: *(*p* < 0.05), **(*p* < 0.01), ***(*p* < 0.001), and ****(*p* < 0.0001).

Abbreviations: CE, cardioembolism; CR, cancer‐related; LAA, large artery atherosclerosis.

### 3.5 Random Forest Feature Selection to Identify Stroke Mechanisms in the Undermined Cause Group

**FIGURE 3:**
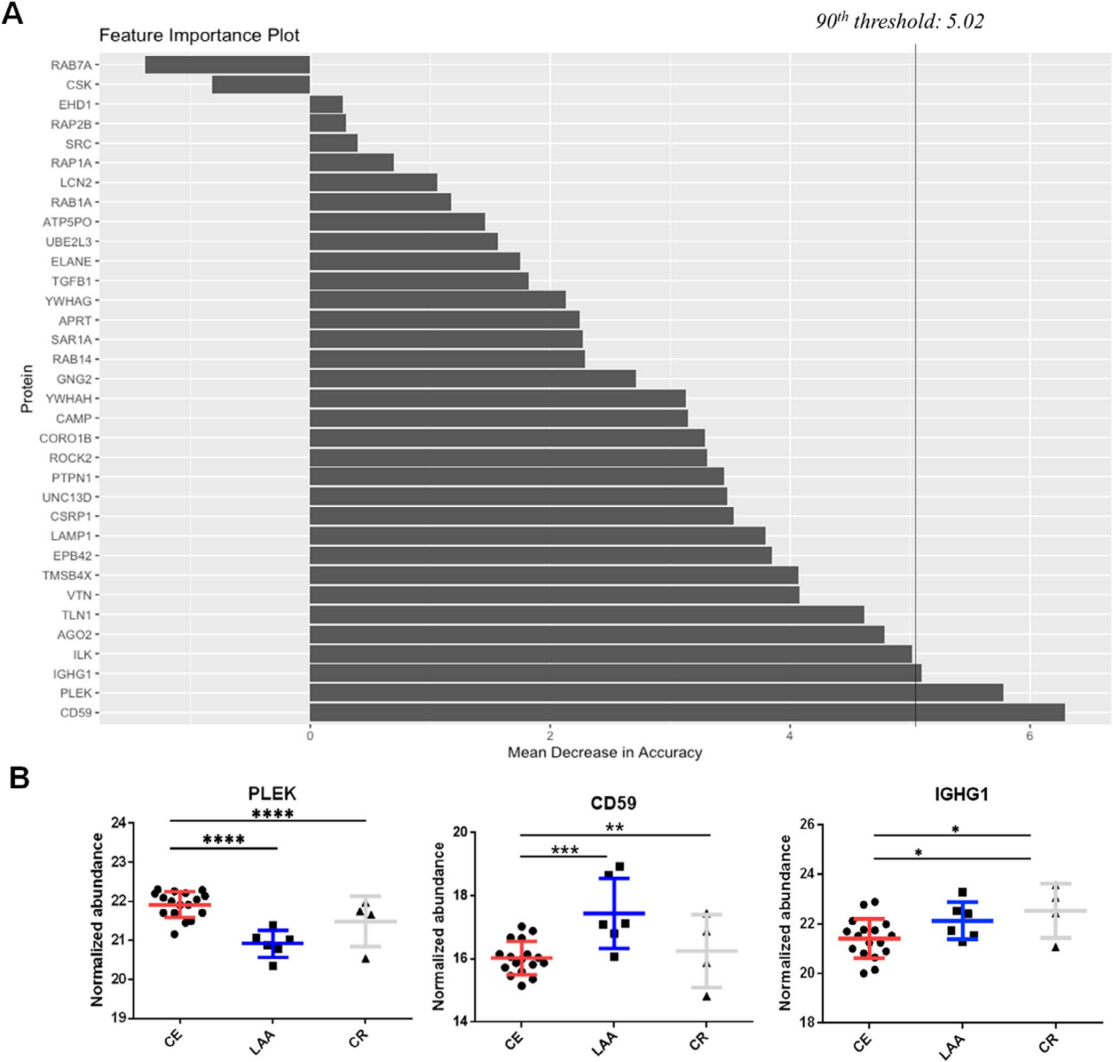
Results of feature selection to predict the UD (undetermined) group. Importance plot after random forest feature selection and hyperparameter tuning using AutoML. We refined the results with 90th percentile, which shows the top 10% of importance scores (threshold: 5.02). The 90th percentile is indicated by a dashed line (A). Scatter plot of the features selected as prediction model. Pleckstrin (PLEK), CD59 glycoprotein (CD59), and Immunoglobulin Heavy Constant Gamma 1 (IGHG1) were specifically upregulated in the CE, LAA, and CR groups, respectively (B). The median and standard deviation of the intensity data are indicated by dashed lines and distinguished by color. The statistical significance are marked with asterisks (*P < 0.05, **P < 0.01, ***P < 0.001, and ****P < 0.0001).

To categorize the stroke mechanisms in the UD group, a combination of the RF machine learning algorithm and deep learning‐based AutoML hyperparameter tuning was employed to construct a prediction model. The intensity data of the final prediction feature candidates were generated and applied to the intensity data of the UD group using DDA analysis. First, the intensity data of the feature candidates were subjected to RF feature selection using 500 trees. Concurrently, the deep learning AutoML was applied to regulate the complexity of the model and to prevent overfitting or underfitting. This analysis yielded 879 models, and the features were evaluated and scored by visualizing the importance plot (Figure 3A) to determine the most reliable features. Thirty candidates were evaluated and ranked according to their importance. The selection process involved refining the list to include only the top 10% most reliable features. As a result, PLEK, CD59, and IGHG1 proteins were chosen (Figure 3B). Proteins in the CE, LAA, and CR groups exhibited statistically significant and specific upregulation, collectively forming a model. The developed model was implemented in a cohort of eight patients belonging to the UD group. The developed model suggested that CE is the most likely cause of ischemic stroke in six patients, and it suggested the CR mechanism in one patient. In one patient, the developed model suggested the LAA mechanism by the developed model, further observation during the follow‐up period confirmed that the patient had AF, which led to the conclusion that CE was the cause of the ischemic stroke (Table S11). Among eight patients during the follow‐up period, the stroke mechanism was confirmed in four patients, and three of them matched the results of the prediction method, indicating a positive predictive value (PPV) of 75.0%, whereas in four cases, the cause remained UD and prediction rates (Tables S11 and S12).

## 4 Discussion

This study conducted a proteomic analysis of patients with ischemic stroke treated with EVT, identified specific biomarkers, and elucidated the underlying biological pathways associated with stroke mechanisms, including LAA, CE, and CR. In CE clots, elevated levels of proteins associated with thrombosis and hemostasis pathways, such as PLEK, ROCK2, TLN1, and RAB14, were detected. In addition, proteins from clots were identified with increased expression in arterial blood of the CE group. The LAA mechanism clots demonstrated elevated levels of proteins such as CD59, LAMP1, and ELANE, which are associated with the ubiquitin‐proteasome pathway and the progression of atherosclerosis. CR clots exhibited high levels of proteins associated with active cancer and immune responses, such as IGHG1 and VTN. These proteins are involved in tumorigenesis and the immune system, including the complement system. Additionally, a machine learning‐based approach was developed to accurately identify stroke mechanisms by analyzing the specific proteins associated with mechanisms. Notably, three proteins, PLEK, CD59, and IGHG1, exhibited precise categorization of the three groups, yielding a 75.0% PPV.

The accurate determination of the underlying cause of ischemic stroke is a fundamental component for secondary prevention [1, 2]. Patients with an UD cause have limitations in choosing appropriate antithrombotic therapy for secondary prevention, leading to an increased risk of stroke recurrence when compared to individuals with a known cause of stroke [18, 19]. Previous studies have attempted to establish a correlation between thrombus composition and stroke etiologies through histopathological and immunohistochemical analyses [3, 4, 5, 6]. Histological analysis was used to assess the composition of blood clots, which typically include RBCs, platelets/fibrin, and leukocytes [3, 4, 5, 6, 20]. Several studies have suggested a correlation between RBC‐rich clots and CE etiology, whereas platelet/fibrin‐rich clots have been linked to LAA etiology [5]. However, contrasting findings have been presented by other studies, suggesting that fibrin‐rich clots are associated with CE, while RBC‐rich clots are associated with LAA [6, 20]. Furthermore, a few studies reported a lower proportion of RBCs in clots from a CR ischemic stroke [6, 13]. However, our study found that there was no significant difference in the compositions of RBCs, fibrin, and leukocytes in clots among the three mechanisms in consistency with previous some studies [4, 6]. Histological analysis showed inconclusive results in terms of identifying stroke mechanisms using clots.

This study identified specific pathways and biomarkers to differentiate ischemic stroke mechanisms using LC–MS/MS analysis and a machine learning method. We identified 260 of the 3726 proteins that were analyzed in the clots. These proteins are categorized into distinct clusters based on their involvement in key biological processes related to stroke mechanisms. Proteins such as PLEK, ROCK2, TLN1, and RAB14 in CE were associated with platelet activation, platelet adhesion, and thrombus formation. Proteins including CD59, LAMP1, and ELAN involved in LAA play crucial roles in the survival, proliferation, and migration of macrophages, potentially influencing the development of atherosclerosis in large arteries. In the CR mechanism, the identified biological processes with IGHG1 and VTN proteins were tumor cell growth and the microenvironment of the tumor cells. Regarding the mechanisms of CE and LAA, the key biological processes observed in this study were similar to those reported in previous studies [21, 22]. However, no study has investigated the biological processes and proteins involved in the CR mechanism.

We developed a new method using machine learning based on RF feature selection following the PPI and validated DEPs. Therefore, we predicted the mechanisms of stroke based on three identified proteins. First, we found that PLEK is a reliable predictor of CE stroke. This protein plays a significant role in thrombus platelet activation, platelet adhesion, and thrombosis, as it is a major target of protein kinase C in platelets [23, 24]. Second, CD59 is an identified protein for predicting the LAA mechanism, as it has a regulatory role in complement membrane attack complex assembly for mediating endothelial damage and foam cell formation of the atherosclerosis plaque. These pathways are involved in the atherosclerotic process in endothelial cells, smooth muscle cells, and inflammatory cells [25, 26, 27]. Finally, IGHG1 was found to be a reliable predictor of CR stroke, known to promote tumor expansion and invasion in multiple malignancies [28, 29, 30]. By applying the developed method to eight patients with strokes of an UD cause, we predicted the underlying mechanisms with an impressive 75.0% PPV.

Distinguishing cell‐derived from blood‐derived proteins in clots is challenging due to significant overlap in protein composition between blood components and cells involved in clotting, compounded by protein modifications and degradation within the clot. Furthermore, especially with samples from biopsies or minimally invasive procedures, limited clot quantities hinder comprehensive validation using traditional biochemical methods like Western blotting and IHC, which require substantial material for multiple target analyses. Consequently, accurately pinpointing protein origin and spatial distribution within clots necessitates employing advanced techniques such as MS‐based spatial proteomics, offering higher sensitivity, multiplexing capabilities, and spatial resolution even with limited samples. This approach is crucial for a more comprehensive understanding of clot composition and dynamics, ultimately facilitating the development of targeted diagnostic and therapeutic strategies.

This study has several limitations. First, the sample size is relatively small. Despite this limitation, we conducted an analysis of various mechanisms, such as cancer– stroke. Furthermore, identified proteins of the arterial blood samples in the occluded arteries matched the proteins found from the clots in the CE mechanism. Second, owing to the limited size of the study, we were unable to evaluate potential confounders such as prior use of antiplatelet agents, anticoagulants, and tPA. In addition, we could not adjust the ordinary *p*‐value using multiple hypothesis testing due to the large inter/intra‐group variation of clot protein level as well as the small sample size. To make up for the lack of statistical power, we performed additional experiments using an orthogonal method to verify the results of the discovery stage. Furthermore, hyperparameter tuning and threshold settings were applied to minimize these errors in the development of the prediction model. Third, the power of the validation study in the UD group is limited because of a small sample size and follow‐up period of the UD group (*n* = 8).

## 5 Conclusions

In conclusion, potential clot protein biomarkers linked to stroke mechanisms such as CE, LAA, and CR have been identified in this study. Furthermore, a methodology was devised to facilitate the discovery of biomarkers specific to various stroke subtypes, including CE, LAA, and CR, utilizing proteomic analysis. Our findings indicate the potential utility of clot analysis in identifying distinct biomarkers linked to the pathophysiology of thrombus formation. This information may help clinicians better differentiate stroke etiologies, particularly in cases with UD causes, such as those having two or more etiologies or embolic stroke of UD source. Furthermore, it could help in choosing optimal antithrombotic or anticoagulation strategies for secondary prevention based on the specific stroke etiologies. This pilot study is based on a small sample size; therefore, more extensive research is needed to validate and establish these correlations in a larger patient population.

## Author Contributions

S.‐B.K., B.‐W.Y., and T.J.K. contributed to the study concept and design. T.J.K., J.W.J., D.H., and Y.J.K. contributed to data analysis. T.J.K., B.‐W.Y., and S.‐B.K. contributed to data collection. T.J.K., J.W.J., D.H., and S.‐B.K. drafted the manuscript. All authors read and approved the manuscript.

## Disclosure

The authors have nothing to report.

Permission to reproduce material from other sources: Authors are responsible for obtaining permission to use any copyrighted material contained in other materials.

## Ethics Statement

This study was approved by the Institutional Review Board (IRB) of Seoul National University Hospital (No. H‐1805‐050‐944 and H‐1803‐078‐931), and the participants provided written informed consent.

## Conflicts of Interest

The authors declare no conflicts of interest.

## Supporting information


Figures S1–S9

Tables S1–S13

Data S1.

Data S2.

## Data Availability

To inquire access to the study data, contact the corresponding author (Sang‐Bae Ko).
