# Supplementary material for: Proteomic Analyses of Clots Identify Stroke Etiologies in Patients Undergoing Endovascular Therapy
Source: CNS Neurosci Ther. 2025 Mar 13;31(3):e70340. doi: 10.1111/cns.70340 (PMC11904956; doi:10.1111/cns.70340)

**Supplementary Figure 1. Flow chart of proteome data processing of differentially expressed proteins identification from DDA analysis and feature selection for prediction by DIA analysis**

**
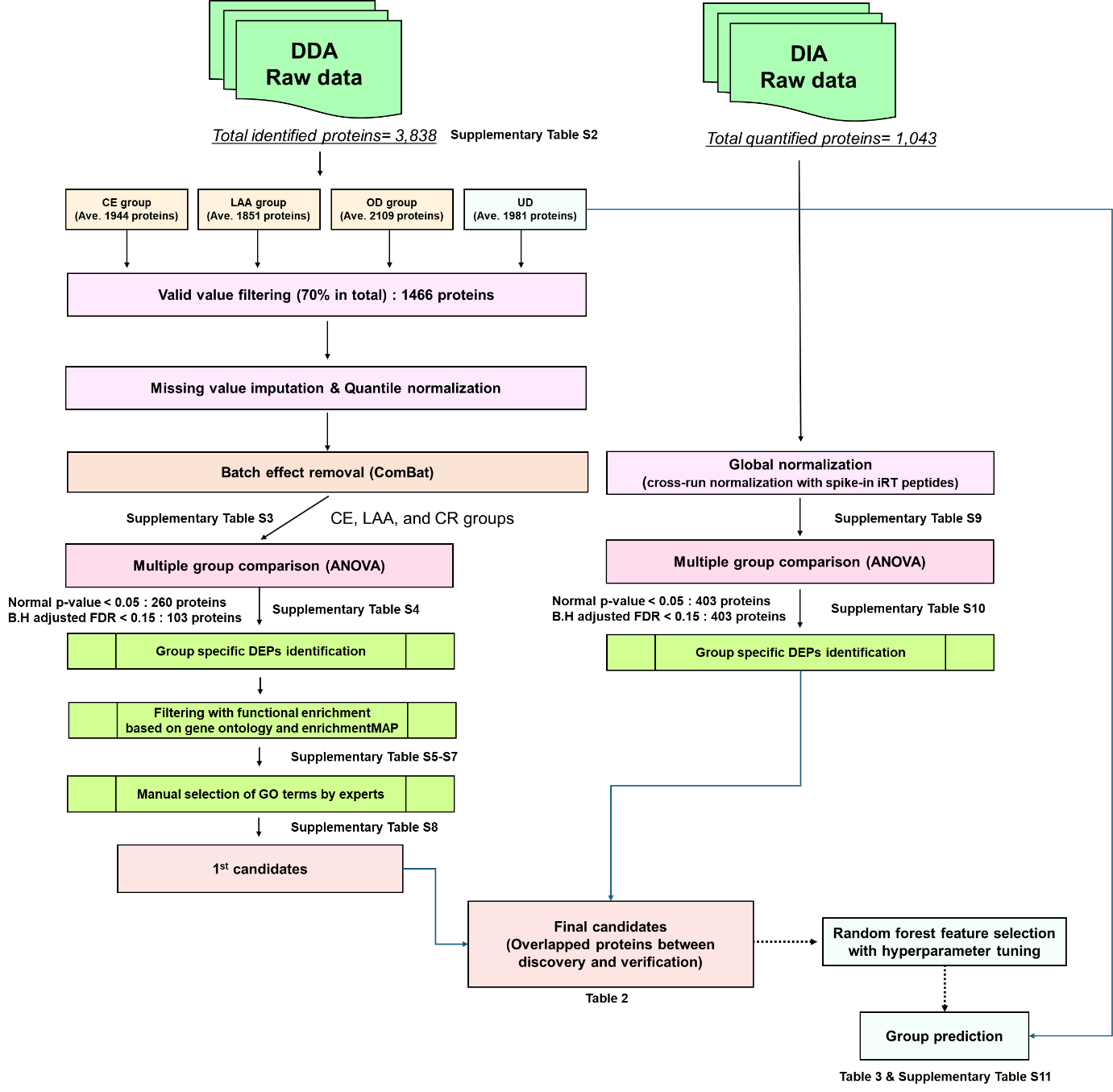
**

During the identification of DEPs, the iBAQ values of CE, LAA, and UD groups were used, and ANOVA multiple comparison tests were performed after quantile normalization. DIA analysis followed an equivalent procedure to identify the DEPs, except for the normalization method. Multiple testing correlation was performed using the Benjamini-Hochberg procedure. Common DEPs from the DDA and DIA analyses with the same expression were applied to random forest feature selection and hyperparameter tuning to predict the UD group from the DDA analysis.

**Supplementary Figure 2. Representative histopathological images (H &E stain) of the clots retrieved after EVT of included patients.**


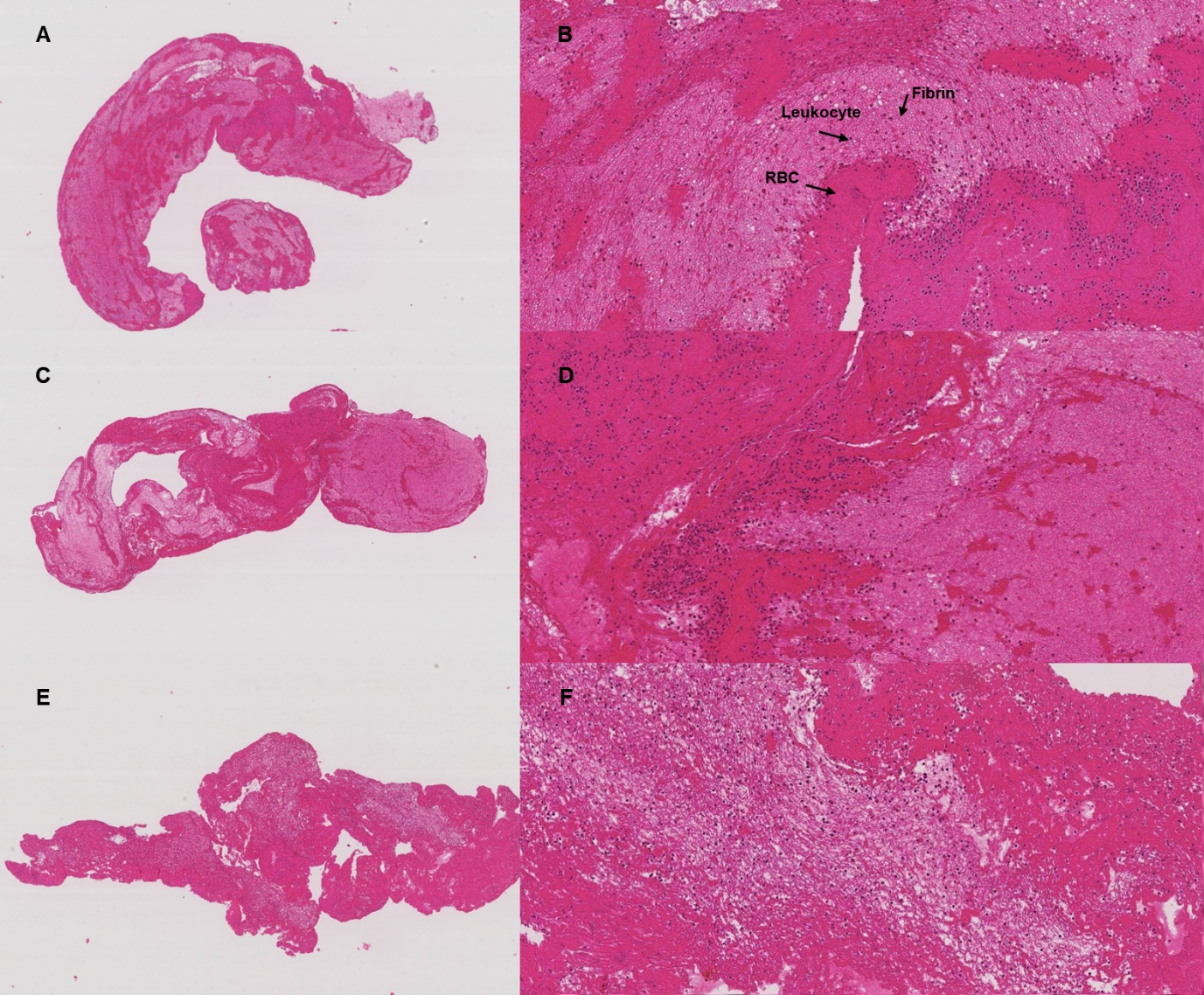


(A) Low-power view of clot from the CE patient (×1), (B) high-power view of from the CE patient (×10), (C) Low-power view of clot from the LAA patient (×1), (D) high-power view of from the LAA patient (×10), (E) Low-power view of clot from the CR patient (×1), and (F) high-power view of from the CR patient (×10)

EVT: endovascular therapy; CE: cardioembolism; LAA: large atherosclerosis; CR: caner related

**Supplementary Figure 3. Overall scheme of analysis.**

**
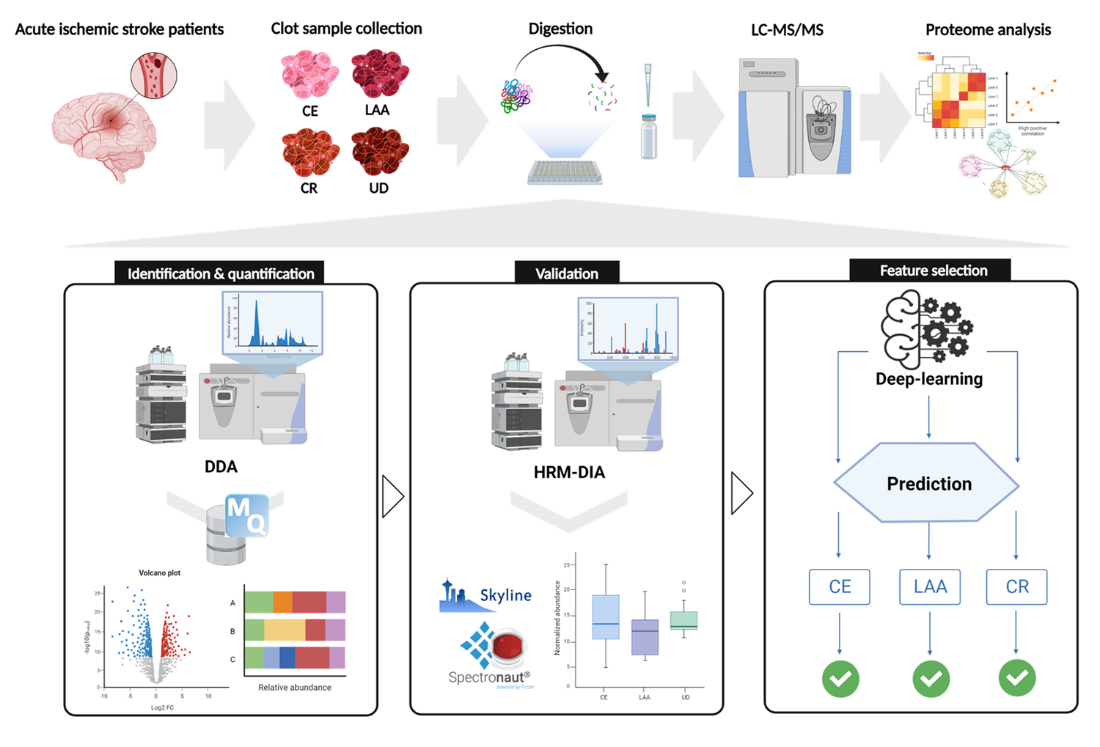
**

This figure was created with Biorender.com and exported under a paid subscription.

**Supplementary Figure 4. LC-MS/MS results of global proteomics analysis of clot samples in discovery stage**


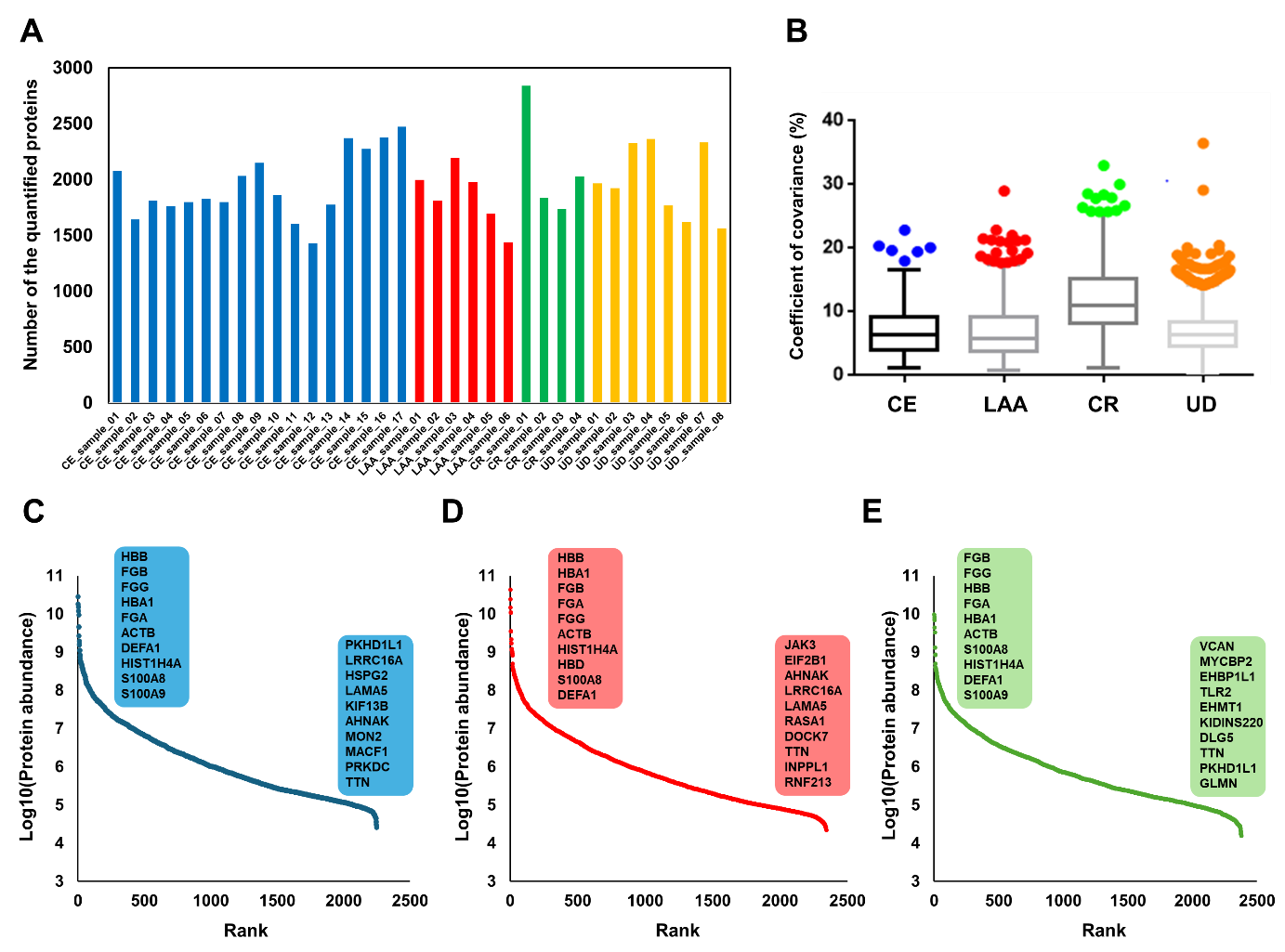


The proteomic features following liquid chromatography-tandem mass spectrometry (LC-MS/MS) analysis. (A) The number of quantified proteins in individual samples. (B) Coefficient of variance in each group. The dynamic range of the protein identification of CE (C), LAA (D), and CR (E) groups according to the descending sort of protein abundance. Proteins are quantified as log10 transformed intensity. Top 10 most abundant proteins and the 10 least abundant proteins were shown.

**Supplementary Figure 5. Overview of global proteome analysis of clot samples from data-dependent acquisition (DDA) analysis**

(A) Venn diagram showing the protein overlap of cardioembolic (CE), large artery atherosclerosis (LAA), and cancer-related stroke (CR) group. The number of proteins is identified in CE (n=17), LAA (n=6), and CR (n=4) samples.

(B) The principal component analysis (PCA) plot of three stroke mechanisms samples.

(C) hierarchical clustering analysis using proteins with ANOVA p-value < 0.05. Three main clusters were identified according to three stroke mechanisms.


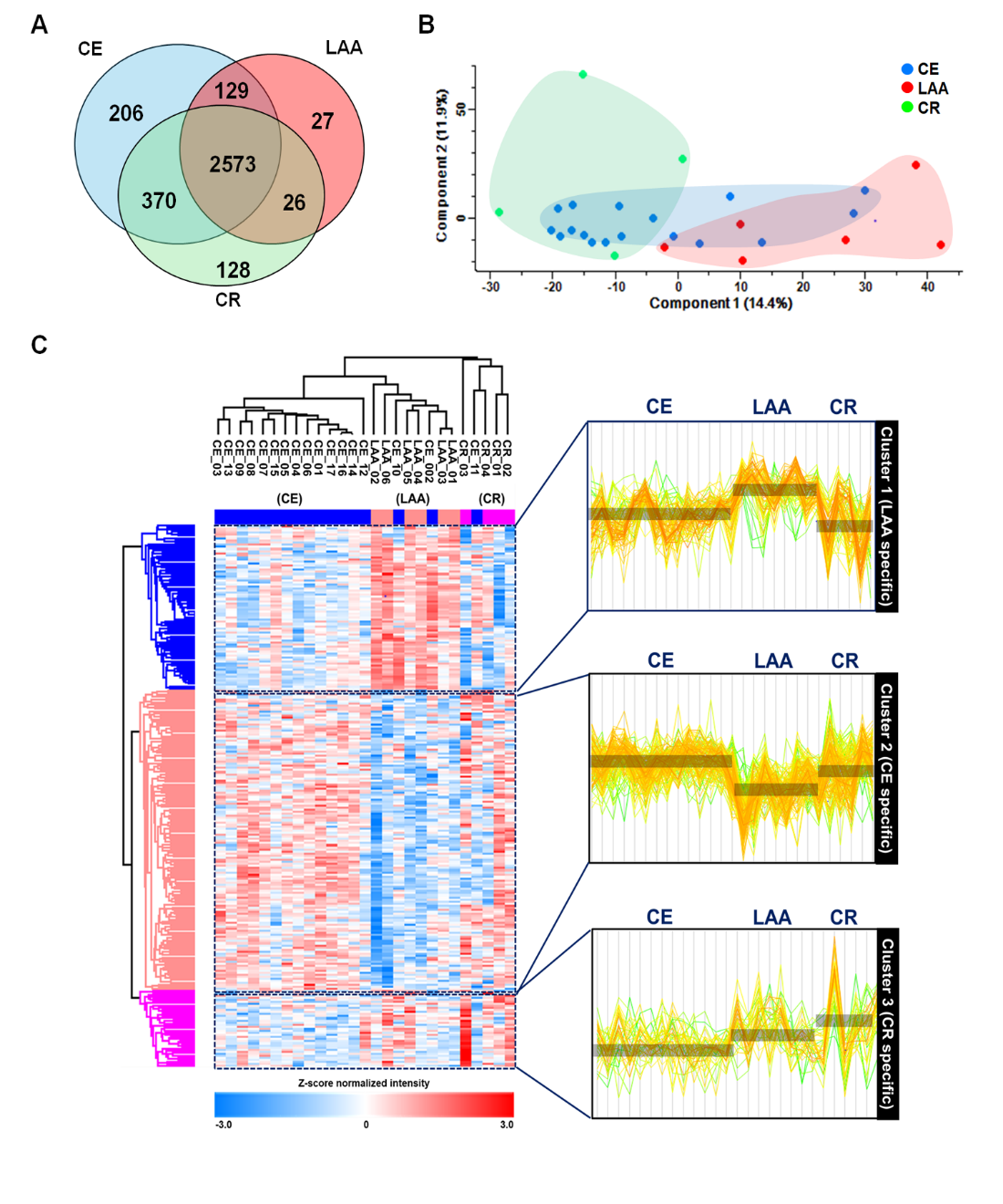


**Supplementary Figure 6.** **Functional analysis based on gene ontology (GO) enrichment and protein-protein interaction (PPI) analysis**

Bubble plots showing top 20 of biological process GO terms (two-sided Fisher’s exact test) of LAA (A), CE (C), and CR (E) groups. The fisher’s exact test p-value < 0.05 is considered statistically significant. Protein-protein networks based on proteins included in the manually selected GO terms in each cluster. PPI networks were generated using 62, 57, and 30 proteins in cluster 1 (B), cluster 2 (D), and cluster 3 (F), respectively. The interactions were evaluated using the BottleNeck ranking method, and the scores were indicated using a colorimetric scheme. Nodes in blue circle indicated main hub proteins.


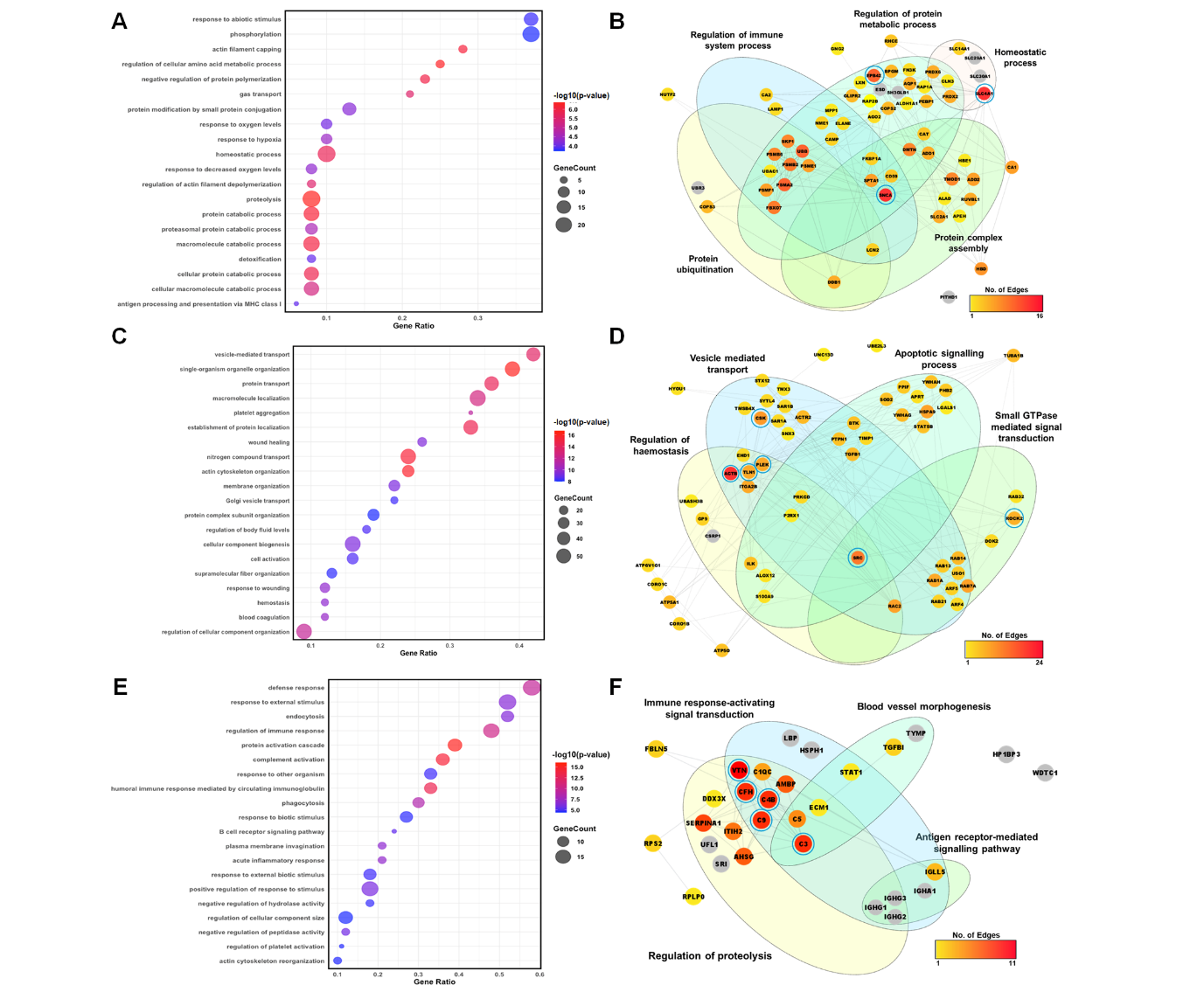


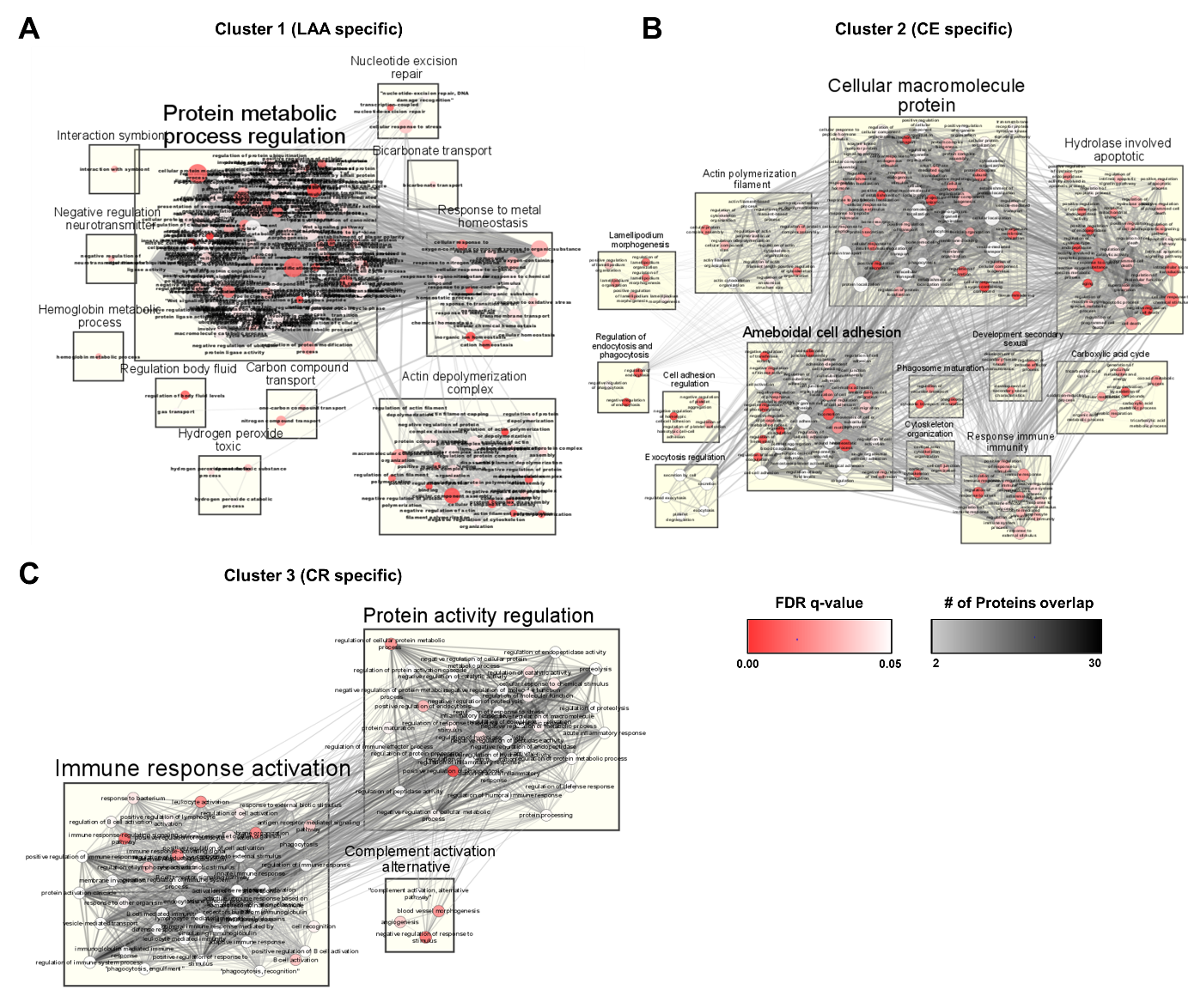


**Supplementary Figure 7. EnrichmentMap analysis using proteins in three stroke mechanism clusters.**

The GO terms were categorized by EnrichmentMap analysis from (A) LAA, (B) CE, and (C) CR group with stringent criteria (FDR q-value < 0.05 and overlap coefficient > 0.5). All nodes describe biological processes from GO analysis with colorimetric scheme of false discovery rate (FDR) q-value. The color and width of edge presents the number of genes overlapping between different GO terms.

**Supplementary Figure 8. Results of verification experiments in clot samples using data-independent acquisition (DIA) analysis**

Hierarchical clustering analysis using proteins with ANOVA P-value < 0.05 in DIA data. Three major clusters were detected based on protein expressions that upregulated in each stroke mechanism groups. Venn diagrams showed the common proteins between candidates selected from discovery stage and proteins in verification stage.


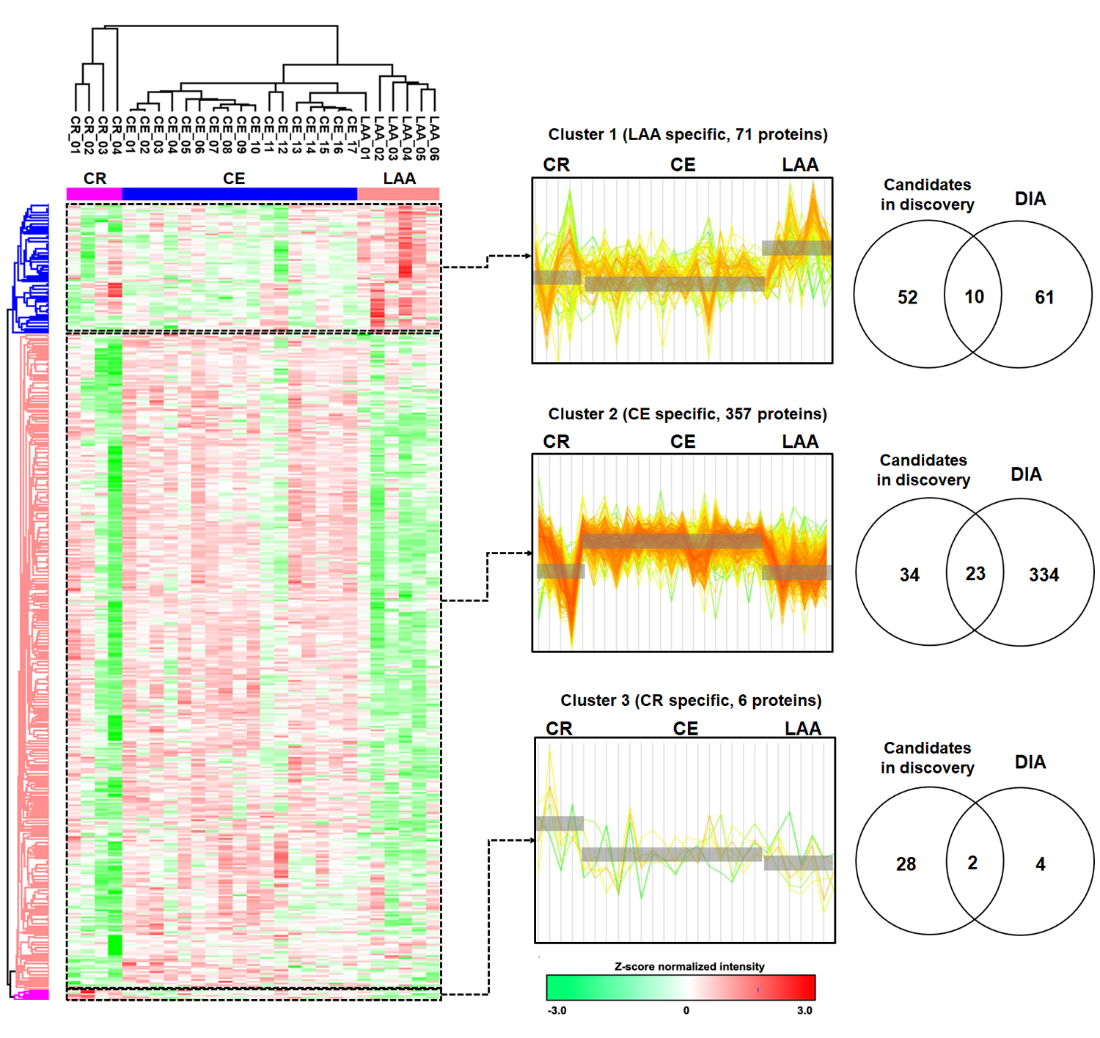


**Supplementary Figure 9. Process for identifying proteins of intra-arterial blood samples in the CE group**

(A) Importance plot after random forest feature selection and hyperparameter tuning using AutoML. We refined the results with 90th percentile, which shows the top 10% of importance scores (threshold: 5.02). The 90th percentile is indicated by a dashed line. This figure was created with Biorender.com and exported under a paid subscription.

(B) Volcano plot showing the differential expression of pre endovascular therapy (EVT) and post EVT (paired t-test). The P < 0.05 is considered statistically significant. Orange filled circle indicated significantly differentially expressed proteins (DEPs). Venn diagram showed the protein overlap between CE specific clusters in discovery and verification using clot samples and DEPs obtained from serum samples.

(C) Expression changes of 5 proteins from pre EVT to post EVT in serum samples. Significance levels (paired t-test) are indicated by asterisks: * P < 0.05.


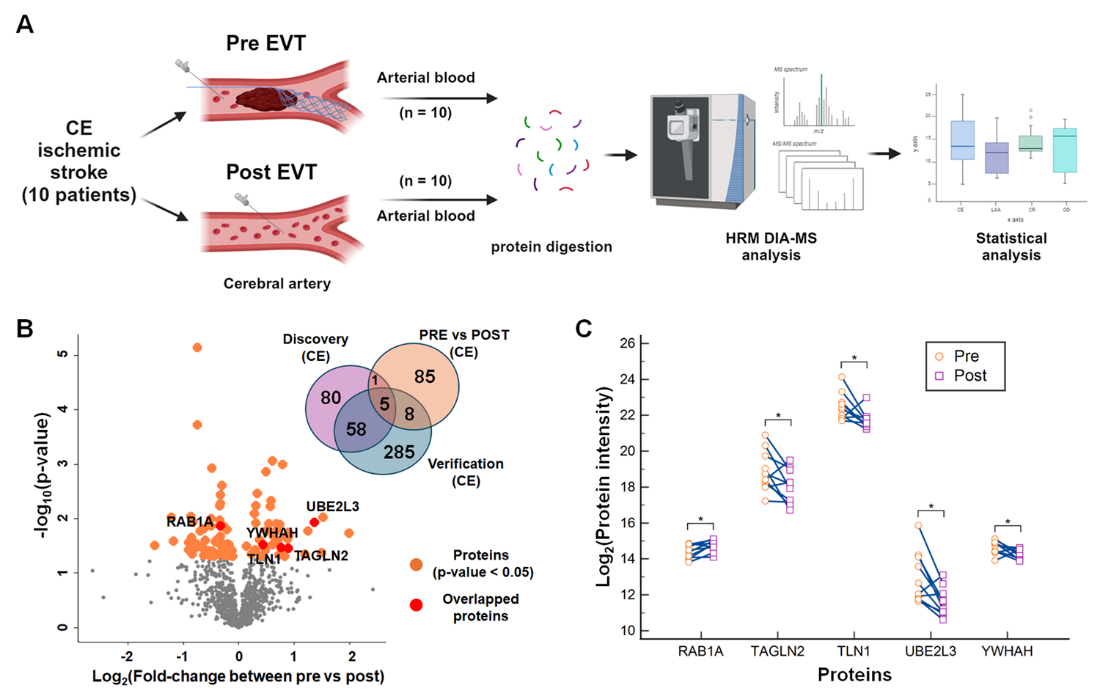

Supplement: Supplementary file 1 — Figures S1–S9 [file CNS-31-e70340-s002.docx]
