# Supplementary material for: Proteomic Analyses of Clots Identify Stroke Etiologies in Patients Undergoing Endovascular Therapy
Source: CNS Neurosci Ther. 2025 Mar 13;31(3):e70340. doi: 10.1111/cns.70340 (PMC11904956; doi:10.1111/cns.70340)
Supplement: Supplementary file 3 — Data S1. [file CNS-31-e70340-s004.docx]

**Supplementary Methods**

**Histological analysis**

The clot that was obtained was immersed in a 10% formalin solution (containing 3.7% formaldehyde) and subsequently embedded in paraffin.^1-3^ Following this step, the clot was cut into 4-μm thick slices and subjected to hematoxylin-eosin (H&E) staining. A SCN400 slide scanner microscope and digital camera (Leica, Germany) were used to capture random photographs of stained slices at 200× magnification. ImageJ software (National Institutes of Health, Bethesda, Maryland) was used for the semi-quantitative analysis of the proportion of RBC and fibrin based on the area.^1-3^ This analysis was conducted by two investigators who were blinded to the patients' clinical data, ensuring objectivity and minimizing bias.

**Samples preparation for proteomic analysis**

Clot samples were collected from different groups, including CE (n = 17), LAA (n = 6), CR (n = 4), and UD causes (n = 8), were subjected to global proteomic analysis. The extraction of these samples was performed by homogenizing them in lysis buffer (4% SDS, 2 mM Tris-(2-carboxyethyl) phosphine, and 0.1 M Tris-HCl at pH 7.4). Protein concentration was determined using a BCA protein assay kit (Thermo Fisher Scientific, MA, USA). The proteins (100 µg) were precipitated using acetone at –20°C overnight. After precipitation,^4^ proteins were digested using a two-step filter-aided sample preparation (FASP) process.^5^ The pellets were resuspended in 40 µL of digestion buffer (2% SDS, 10 mM TCEP, and 50 mM CAA in 0.1 M Tris at pH 8.0). The solutions were transferred into a 30 K Amicon filter (Millipore, MA, USA) and subjected to centrifugation at 14,000 × g at 25°C before washing with UA solution (8 M urea in 0.1 M Tris at pH 8.5). Subsequently, the solution was replaced with a 50 mM HEPES buffer, and the protein sample was subjected to digestion using a trypsin/LysC mixture at a protein-to-protease ratio of 100:1) overnight at 37°C. The peptides were eluted by centrifugation and rinsed with 50 mM HEPES. The second digestion involved the incubation of trypsin with the protein sample at 37°C for 3 h with an enzyme-to-protein ratio of 1:1,000. The concentration of digested peptides was determined using a tryptophan fluorescence assay. The samples were acidified using a 10% trifluoroacetic acid. For discovery experiments, peptides were desalted and fractionated on a homemade styrene divinylbenzene reversed-phase sulfonate (SDB-RPS)-StageTips^6^ by high-pH reverse-phase using a stepwise gradient of acetonitrile (40, 60, and 80%) in 1% ammonium hydroxide. For verification experiments, digested peptides were desalted using homemade C18-stageTips, following a previously described method.^7,8^ For intra-arterial serum samples, proteins were digested using a two-step FASP process described above. To remove contaminant, 10 ug of digested peptides were desalted according to protocol described above. All peptide samples were ultimately subjected to vacuum drying and subsequently stored at –80°C until further analysis.

**Liquid chromatography-tandem mass spectrometry (LC-MS/MS) analysis**

Proteome discovery analysis was performed by data-dependent acquisition (DDA) using the prepared clot samples, and validation was performed using independent samples by data-independent acquisition (DIA). The mass spectrometry (MS) analyses were performed utilizing quadrupole Orbitrap mass spectrometers, namely the Q-Exactive HF-X (DDA) and Orbitrap Exploris 480 (DIA), manufactured by Thermo Fisher Scientific, MA, USA. These instruments were coupled with an Ultimate 3000 RSLC system (Dionex, Sunnyvale, CA, USA), which consisted of EASY-Spray^TM^ LC columns and an electrospray ionization source. Before injecting the samples, the peptide samples that had been dried were reconstituted in solvent A, which consisted of 2% acetonitrile and 0.1% formic acid. The peptide samples were subjected to separation using a two-column system. This system comprised of a trap column (300 µm I.D. × 0.5 cm, C18 3 µm, 100 Å) to eliminate any potential contaminants, and an analytical column (75 µm I.D. × 50 cm, C18 1.9 µm, 100 Å). The separation process involved a 90-minute gradient from 8% to 30% Solvent B (composed of 80% acetonitrile and 0.1% formic acid) at a flow rate of 300 nL/min. The temperature of the column heater was set to 60°C. In positive mode, the spray voltage was set to 2.0 kV, while the heated capillary temperature was set to 320°C.

For the DDA analysis, mass spectra were obtained using a Q-Exactive HF-X instrument in data-dependent mode, employing the top 15 methods. The precursor ions were scanned within a mass range of 300-1,650 m/z, with a resolution of 70,000 at 200 m/z. High-energy collisional dissociation (HCD) scans were performed at a resolution of 17,500. The HCD peptide fragments were acquired using stepped HCD collision energies of 25, 27, and 29. The maximum ion injection times for the survey and MS/MS scans were set at 25 ms and 50 ms, respectively.

For the DIA experiments, the full scan method implied scan ranges of 300–1,600 m/z with a resolution of 60,000 and an AGC target of 3 × 10^6^ at a 25 ms injection time. The DIA scan consisted of 23 DIA isolation windows at 40 ms and was acquired at a resolution of 35,000. An automatic injection time (AGC)^9^ was applied.

**Spectral library construction for DIA analysis**

To construct a peptide library for matching between runs^10^ in discovery experiments (DDA), pooled clot samples were digested using a 2-step FASP procedure, as described above. The peptides that had undergone digestion were desalted and purified through Oasis HLB solid-phase extraction (SPE). To obtain a comprehensive dataset, 100 µg of the purified peptides were separated into fractions using an Agilent 1260 bioinert HPLC system (Agilent, CA, USA) with an analytical column measuring 4.6 × 250 mm and containing 5-µm particles. A high-pH reversed-phase peptide fractionation technique was employed to separate peptides. The separation process was carried out at a flow rate of 0.8 mL/min, using solvent A (15 mM ammonium hydroxide in water) and solvent B (15 mM ammonium hydroxide in 90% acetonitrile). A total of 96 fractions were collected at one-minute intervals and subsequently combined into 24 non-contiguous fractions. These fractions were then dried using a vacuum centrifuge and stored at a temperature of -80°C until further analysis using LC-MS/MS.

**DDA data processing for discovery experiments**

The raw DDA files were processed using MaxQuant (version 1.6.1.0). Tandem mass spectra were searched against the *Homo Sapiens* reference in the UniProt database (December 2014; 88,657 entries) and contaminants using Andromeda. Primary searches were performed with 6 ppm precursor ion tolerance for total protein level and 20 ppm for MS/MS ion tolerance. Variable modifications, such as N-terminal acetylation of proteins and oxidation on methionine, and a fixed modification, carbamidomethylation on cysteine, were set for the database search. The enzyme was configured to undergo a complete tryptic digestion, and the length of peptides was determined based on six amino acids with two instances of missed cleavages. The false discovery rate (FDR) for identifying peptides and proteins was set at 1%. In order to optimize the number of quantification events across the samples, a matching process between runs was conducted, utilizing the pooled clot sample as a reference library. The intensity-based absolute quantification (iBAQ) value was calculated as the sum of the identified peptide intensities for the proteins divided by the number of theoretical trypsin cleavage sites in the protein sequence. This allows for a relative measure of protein abundance suitable for comparing proteins across different samples under different experimental conditions. The mass spectrometry DDA data have been deposited to the ProteomeXchange Consortium via the PRIDE^11^ partner repository with the dataset identifier PXD050508. Annotated MS/MS spectra can be accessed through MS-viewer^.12^

(<https://msviewer.ucsf.edu/prospector/cgi-bin/mssearch.cgi?report_title=MS-Viewer&search_key=mxemj51nun&search_name=msviewer>) with the following search keys: mxemj51nun

**DIA data processing**

All DIA data of independent samples from clot and intra-arterial serums were analyzed using Spectronaut (version 15; Biognosys) and the raw files were converted to htrm format using GTRMS converted embedded in Spectronaut software. For general DIA analysis with the highest data completeness, DIA files were extracted from a direct DIA library built from the DIA data (DDA-independent library).^13^ Pulsar embedded in Spectronaut (version 15) was used to generate a directDIA library against UniprotKB Human protein sequence database (2021 july, 101,104 entries). In brief, the settings for Pulsar and library generation were as followings: Trypsin/P as specific enzyme; peptide length from 6 to 52; max 2 missed cleavages; Carbamidomethyl on C as fixed modification; Oxidation on M and Acetyl at protein N-terminus as variable modifications; FDRs at PSM, peptide and protein level all set to 0.01; minimum fragment relative intensity 1%; 3–6 fragments kept for each precursor. MS1 and MS2 tolerance were set as dynamics. The mProphet algorithm was used to calculate the FDR of peptide precursors and protein levels below 1%.^14^ The quantification source was also acquired at the protein level by applying a criterion of q-value < 0.01, and this setting was used for subsequent analyses. The mass spectrometry DIA data have been deposited to the ProteomeXchange Consortium via the PRIDE^14^ partner repository with the dataset identifier PXD050730.

**Statistical analysis**

Baseline characteristics and clinical information were analyzed descriptively. The normality test was performed using Kolmogorov–Smirnov test or the Shapiro–Wilk test based on the number of cases. Nominal data were expressed as frequency (percentage), normally distributed continuous variables were expressed as mean ± standard deviation, and non-normally distributed continuous variables were expressed as median and interquartile range. To assess differences in baseline characteristics, categorical variables were evaluated using Pearson’s χ^2^ test or Fisher’s exact test, as appropriate. Continuous variables were compared using the Kruskal–Wallis test or one-way analysis of variance (ANOVA). Analyses of clinical information were conducted using SPSS (version 27.0; IBM Statistics, Armonk, NY, USA). Data pre-processing and differentially expressed proteins (DEPs) identification were performed using Perseus software (version 1.6.15.0). For relative quantification in DDA or DIA analyses, we first used the iBAQ value from the discovery stage DDA data, which were log2 transformed to be normally distributed. The proteins that were not identified at over 70% in at least one group were filtered before quantification and missing values were replaced by the normal distribution imputation. After quantile normalization, batch effect remove was performed by ComBat. For a multiple group comparison, ANOVA test was performed. Subsequently, Z-normalization of the normalized protein abundance was specifically applied only to proteins that met the ANOVA p-value < 0.05 criteria. This selective Z-normalization was performed solely for the purpose of hierarchical clustering in terms of Euclidean distance with an average link.

For statistical analysis of the DIA data, protein intensity was calculated by Spectronaut from the average of the top3 peptide. Default settings of quantification were applied, with global normalization enabled. Spectronaut incorporates a robust normalization algorithm that corrects for systematic variations in signal intensity across different LC-MS runs. This global normalization process aims to equalize the overall protein abundance across all samples, mitigating technical variations introduced during sample handling, LC separation, and mass spectrometry analysis. For serum samples of arterial bloods, paired t-test was conducted using Perseus software. All statistically significant values were *P-value* < 0.05. The Benjamini-Hochberg procedure was applied to calculate the False Discovery Rate (FDR) and the adjusted p-value (q-value).

**Bioinformatics analysis**

To investigate the functional characteristics of the DEPs identified through ANOVA multiple comparison, a gene ontology (GO) analysis was performed to explore the biological or physiological processes associated with the DEPs using the DAVID tool (version 2023q2).^15^ DEPs were independently analyzed based on their protein expression patterns within each group to determine their unique functions. Additionally, the GO terms with *P* < 0.05 were filtered. The terms were further filtered by applying a significance threshold of a *P* < 0.05. Protein-protein interaction (PPI) network analysis was conducted using Cytoscape software (version 3.9.1), and the interaction database was accessed through the STRING^16^ (version 10) database. Hub proteins identification was performed by BottleNeck method in cytoHubba.^17^ Finally, we extracted protein concentration information from the "Blood Proteins" dataset within the Human Protein Atlas (<https://www.proteinatlas.org/humanproteome/blood>) (PMID:25613900). This dataset provides comprehensive information on the abundance of proteins typically found in human blood. By incorporating this external information into our analysis pipeline, we were able to compare the relative abundance of proteins identified in our clot proteomic data with their typical concentrations in blood.

**References**

1. S Staessens, S Fitzgerald, T Andersson, et al. Histological stroke clot analysis after thrombectomy: technical aspects and recommendations. Int J Stroke. 2020 Jul;15(5):467-476.
2. T Boeckh-Behrens, M Schubert, A Förschler, et al. The impact of histological clot composition in embolic stroke. Clin Neuroradiol. 2016;26(2):189-97.
3. SK Kim, W Yoon, TS Kim, HS Kim, TW Heo, MS Park. Histologic analysis of retrieved clots in acute ischemic stroke: correlation with stroke etiology and gradient-echo MRI. AJNR Am J Neuroradiol. 2015;36(9):1756-62.
4. AMJ Crowell, MJ Wall, AA Doucette. Maximizing recovery of water-soluble proteins through acetone precipitation. Anal Chim Acta. 2013;24;796:48-54.
5. Wiśniewski, J. R. Quantitative Evaluation of Filter Aided Sample Preparation (FASP) and Multienzyme Digestion FASP Protocols. Anal Chem. 2016;17;88(10):5438-43.
6. SH Kong, JH Lee, JM Bae, et al. In-depth proteomic signature of parathyroid carcinoma. Eur J Endocrinol. 2023;5;188(4):385-394.
7. H Lee, K Kim, J Woo, et al. Quantitative Proteomic Analysis Identifies AHNAK (Neuroblast Differentiation-associated Protein AHNAK) as a Novel Candidate Biomarker for Bladder Urothelial Carcinoma Diagnosis by Liquid-based Cytology. Mol Cell Proteomics. 2018;17(9):1788-1802.
8. JE Kim, D Han, JS Jeong, et al. Multisample Mass Spectrometry-Based Approach for Discovering Injury Markers in Chronic Kidney Disease. Mol Cell Proteomics. 2021;20:100037.
9. R Bruderer, OM Bernhardt, T Gandhi, et al. Extending the Limits of Quantitative Proteome Profiling with Data-Independent Acquisition and Application to Acetaminophen-Treated Three-Dimensional Liver Microtissues. Mol Cell Proteomics. 2015;14(5):1400-10.
10. N Nagaraj, NA Kulak, J Cox, et al. System-wide perturbation analysis with nearly complete coverage of the yeast proteome by single-shot ultra HPLC runs on a bench top Orbitrap. Mol Cell Proteomics. 2012;11(3):M111.013722.
11. Y Perez-Riverol, J Bai, C Bandla, et al. The PRIDE database resources in 2022: a hub for mass spectrometry-based proteomics evidences. Nucleic Acids Res. 2022;7;50(D1):D543-D552.
12. PR Baker, RJ Chalkley. MS-viewer: a web-based spectral viewer for proteomics results. Mol Cell Proteomics. 2014;13(5):1392-6.
13. R Lou, Y Cao, S Li, et al. Benchmarking commonly used software suites and analysis workflows for DIA proteomics and phosphoproteomics. Nat Commun. 2023;6;14(1):94.
14. L Reiter, O Rinner, P Picotti, et al. mProphet: automated data processing and statistical validation for large-scale SRM experiments. Nat Methods. 2011;8(5):430-5.
15. X Jiao, BT Sherman, DW Huang, et al. DAVID-WS: a stateful web service to facilitate gene/protein list analysis. Bioinformatics. 2012;28(13):1805-6.
16. D Szklarczyk, AL Gable, D Lyon, et al. STRING v11: protein–protein association networks with increased coverage, supporting functional discovery in genome-wide experimental datasets. Nucleic Acids Res. 2019;8;47(D1):D607-D613.
17. CH Chin, SH Chen, HH Wu, et al. cytoHubba: identifying hub objects and sub-networks from complex interactome. BMC Syst Biol. 2014;8 Suppl 4(Suppl 4):S11.
