# Supplementary material for: Proteomic Analyses of Clots Identify Stroke Etiologies in Patients Undergoing Endovascular Therapy
Source: CNS Neurosci Ther. 2025 Mar 13;31(3):e70340. doi: 10.1111/cns.70340 (PMC11904956; doi:10.1111/cns.70340)
Supplement: Supplementary file 4 — Data S2. [file CNS-31-e70340-s001.docx]

**Supplementary Results**

**Potential blood protein markers related to stroke mechanisms**

To explore whether our findings could be used as potential blood protein markers are associated with stroke mechanisms, we further performed HRM-DIA experiments using arterial blood from CE stroke patients during pre EVT or post EVT periods. We collected 10 pre-EVT serum samples and 10 post-EVT serum samples from 10 patients with CE mechanism (Supplementary Figure 9A). A total of 1050 proteins were quantified in all serum samples using HRM-DIA approach. We observed that 37% proteins (389 proteins) were commonly identified in verification data obtained from DIA methods. To detect changes in the serum proteome between pre and post EVT, we performed paired t-test analysis and identified a total of 99 differentially expressed proteins with *P*-value < 0.05 (Supplementary Figure 9B and Supplementary Table S13). Further comparison of the proteins included in CE-specific clusters in discovery and verification phase with the DEPs identified in serum samples indicated that five proteins also showed altered protein expression levels between pre and post EVT (Supplementary Figure 9C). Especially, we found that RAB1A, TLN1, UBE2L3, and YWHAH were included in final blood protein candidates related to CE mechanism. Identified proteins from arterial blood were found to have increased expression in the clots of the CE mechanism group.
